# Targeting tumor-associated macrophages in gastric cancer progression and therapy: insights from molecular mechanisms to therapeutic applications

**DOI:** 10.3389/fphar.2025.1549694

**Published:** 2025-06-17

**Authors:** ChengTao Wan, Jie Deng, Yu Zhu, Li Wan, Linyue Xu, Qiuyan Chen, Can Zou, Ju Huang

**Affiliations:** ^1^ Department of Gastroenterology, Jian Yang Hospital of Traditional Chinese Medicine, Chengdu, Sichuan, China; ^2^ The General Hospital of Western Theater Command, Chengdu, Sichuan, China; ^3^ School of Sports Medicine and Health, Chengdu Sport University, Chengdu, Sichuan, China; ^4^ Department of Oncology, Hospital of Chengdu University of Traditional Chinese Medicine, Chengdu, Sichuan, China

**Keywords:** gastric cancer, tumor-associated macrophage, tumor microenvironment, tumor progression, immunotherapy

## Abstract

Gastric cancer (GC) is the fifth most common malignant tumor that imposes heavily public health burdens worldwide. Systemic therapies for gastric cancer (GC), such as chemotherapy, targeted therapy, and immunotherapy, have undergone significant advancements. Nevertheless, the extensive application of anti-cancer agents has resulted in an increasing array of challenges related to drug resistance, presenting a substantial barrier in GC treatment. Tumor-associated macrophages (TAMs) as essential immunomodulators within the tumor immune microenvironment (TIME) of GC, providing novel therapeutic targets due to their capacity for plasticity in reaction to environmental signals. They create a complex network of communication with various immune and stromal cell types, thereby contributing to the immunosuppressive nature of the TME in GC. In this review, we establish the map of the origin and polarization of macrophages in GC. During the process of carcinogenesis, macrophages undergo dynamic phenotypic transitions. Additionally, the interactions between TAMs and tumor cells significantly influence the progression of GC, affecting tumor growth, metastasis, angiogenesis, and drug resistance. Furthermore, this intricate immunomodulatory axis notably enhances resistance to immunotherapy, suggesting that targeting TAMs presents substantial therapeutic opportunities for patients with GC. Approaches such as TAM elimination, TAM repolarization, and CAR-M therapy have been validated in numerous studies. We also elaborate on the challenges faced by the development of targeting TAMs, which may provide innovative perspectives on the GC treatment.

## 1 Introduction

Gastric cancer (GC) ranks as the fifth most prevalent malignant tumor and is the fifth leading cause of cancer-related mortality globally based on the GLOBOCAN 2022 report (Bray et al., 2024). It was estimated that approximately 968,000 new cases and 65,900 deaths of GC patients worldwide in 2022 (Bray et al., 2024). The combination of chemotherapy and molecular targeted therapy has clearly extended overall survival time and enhanced the quality of life for GC patients with advanced stages (Guan et al., 2023). However, the widespread use of anti-cancer agents encounters an increasing number of challenges related to drug resistance, which presents a significant obstacle in clinical oncology (Liu J. et al., 2024).

The pathogenesis of GC is closely linked to immune cells and various types of mesenchymal stromal cells within the tumor microenvironment (TME) (Yasuda and Wang, 2024). While immunotherapy has emerged as a potent clinical approach for the treatment of cancer, its clinical efficacy is limited in the context of advanced GC (Yu Y. et al., 2023). Moreover, there are significant differences in immune responses among individuals. These unfavorable therapeutic responses significantly impede the prognosis of GC patients, which await further investigations to prompt the understanding of the spatial arrangement and functional network within the tumor immune microenvironment (TIME) of GC (Xu et al., 2023).

The TIME comprises a variety of non-tumor stromal cells that play a crucial role in GC progression and immune evasion (Yu Y. et al., 2023; Huang et al., 2024a). It contains various immune cells, such as natural killer (NK) cells, cancer-associated fibroblasts (CAFs), Myeloid-Derived Suppressor Cells (MDSCs), dendritic cells (DCs), tumor-associated macrophages (TAMs), cytotoxic T lymphocytes (CTLs), and regulatory T (Treg) cells, along with the extracellular matrix (ECM) and various immunosuppressive cytokines (Yasuda and Wang, 2024; Mou et al., 2023), providing novel therapeutic targets for cancer therapy. Within the TIME, macrophages possess the ability to mediate vascular damage and induce tumor necrosis, as well as stimulate tumor resistance mechanisms mediated by innate or adaptive lymphocytes (Mantovani et al., 2022). Conversely, in the context of progressed tumor masses, macrophages transform into TAMs, which facilitate malignant progression and immune evasion by modulating TIME (Nasir et al., 2023). Additionally, they represent a significant target for contemporary checkpoint blockade immunotherapy due to their expression of inhibitory counter-receptors, such as programmed cell death ligand 1 (PD-L1), B7-1 and B7-2, which trigger immunosuppressive activity by binging to PD-1 and cytotoxic T lymphocyte-associated antigen-4 (CTLA-4) (Zhang H. et al., 2023; Sharma et al., 2024). The dual potential of macrophages in cancer progression has underscored their therapeutic promising for immunotherapy. In this review, we elaborate on the properties and dynamic transitions of macrophages in TIME of GC. Moreover, we highlight the crosstalk between TAMs and GC cells, emphasizing the essential role of TAMs in cancer progression and the critical modulations of TIME, as well as elucidating current therapeutic regimes by targeting TAMs in the field of GC, which may pave the way for the translation of TAMs-based immunotherapy into clinical course.

## 2 Characteristics and functions of macrophages in the TME of GC

### 2.1 The origin and dynamic transitions of macrophages in GC

**FIGURE 1:**
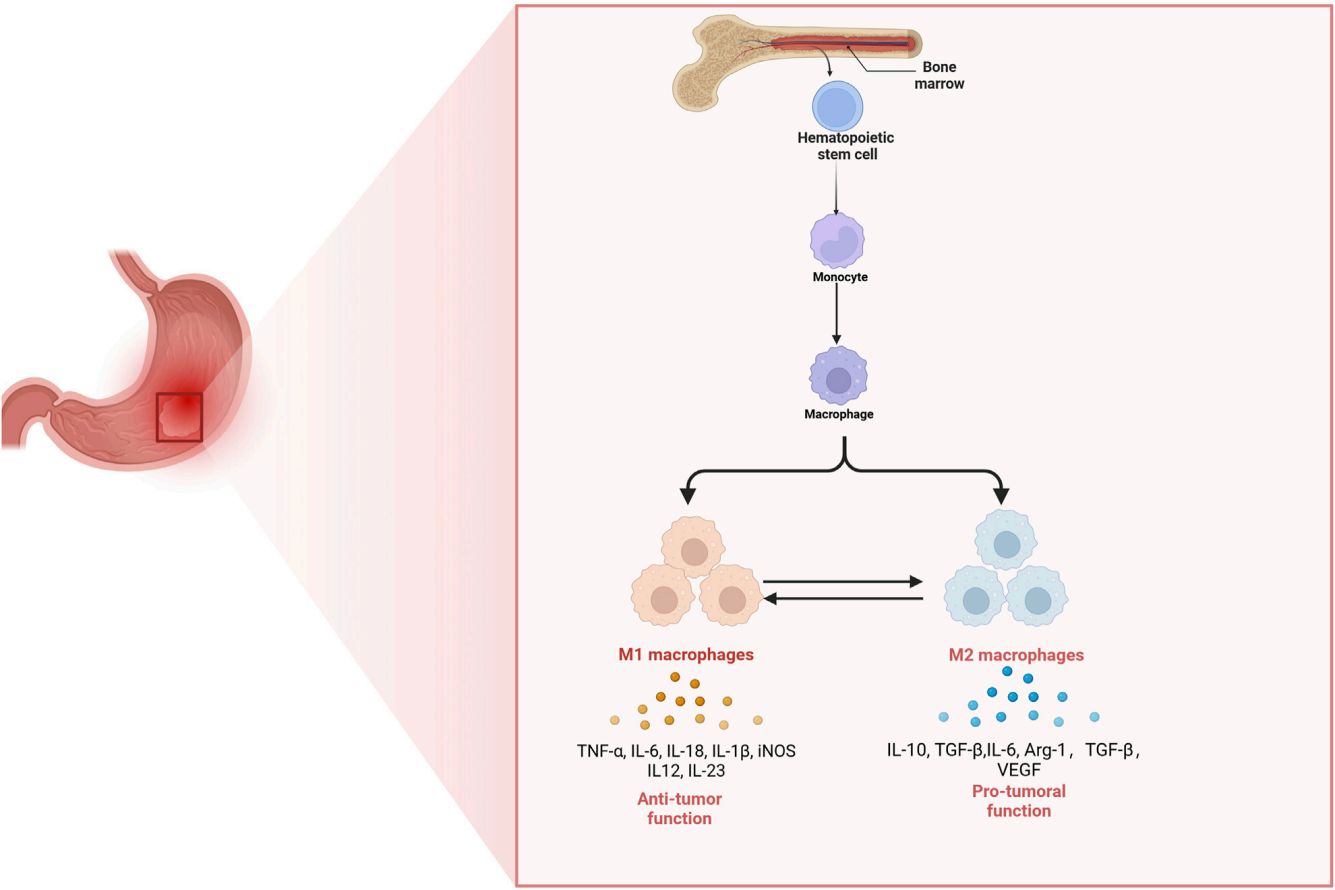
The origin of tumor-associated macrophages and their functions in gastric cancer. Hematopoietic stem cells in the bone marrow differentiate into monocytes, which subsequently migrate to tissues and develop into macrophages. In the gastric tumor microenvironment, macrophages can polarize into either M1 or M2 phenotypes. M1 macrophages secrete pro-inflammatory cytokines (TNF-α, IL-6, IL-18, IL-1β, iNOS, IL-12, and IL-23) and exhibit anti-tumor functions. In contrast, M2 macrophages produce anti-inflammatory and tumor-promoting factors (IL-10, TGF-β, IL-6, Arg-1, and VEGF) and support tumor growth and immune suppression. The balance between M1 and M2 polarization shapes the immune landscape and progression of gastric cancer.

Macrophages exhibit a diverse array of profiles that reflect their inherent plasticity, a characteristic crucial for responding to various danger signals and preserving tissue homeostasis across human organs (Nobs and Kopf, 2021). They are long-lived and recognized as the most versatile cells within significant functional diversity (Wynn et al., 2013). Resident macrophages play crucial roles in multiple aspects of biological progresses, encompassing organ development, tissue homeostasis, and host inflammatory responses against pathogens. During these adaptive processes, macrophages primarily originate from monocyte reservoirs, which are mainly derived from hematopoietic stem cells located in the bone marrow (Guilliams et al., 2018). These macrophages exhibit various tissue-specificities including Osteoclasts, Kupffer cells, Microglia (Locati et al., 2020). However, under some pathological conditions, these homeostatic functions mediated by macrophages can be compromised by ongoing insults, resulting in various disease states, such as obesity, neurodegenerative disorders and cancer (Razi et al., 2023), which suggest that macrophages constitute a highly diverse group of cells that constantly modify their functional states in response to alterations in tissue physiology or environmental cues (Razi et al., 2023) (Figure 1).

In the context of GC, macrophages can be broadly categorized into two opposing groups: classically activated macrophages (M1-like) and alternatively activated macrophages (M2-like) (Li M. et al., 2023). M1 macrophages are typically induced by granulocyte-macrophage colony-stimulating factor (GM-CSF), interferon-gamma (IFN-γ) and lipopolysaccharide, which significantly secret pro-inflammatory factors such as tumor necrosis factor-alpha (TNF-α), interleukin-12 (IL-12), IL-18, IL-1β, and inducible nitric oxide synthase (iNOS) along with large amounts of intracellularly nitric oxide (NO) production (Yunna et al., 2020). M1 macrophages play a crucial role in exerting anti-tumor effects by mediating phagocytosis and antibody-dependent cell-mediated cytotoxicity to eliminate tumor cells (Mantovani et al., 2004; Mantovani et al., 2017). Additionally, M2 macrophages are generally activated by factors such as colony-stimulating factor 1 (CSF1) transforming growth factor-beta (TGF-β), IL-4, IL-10, IL-13, prostaglandin E2 (PGE2) and parasites or fungi. M2 macrophages have the ability to secrete a diverse array of complex cytokines including IL-1β, IL-6, IL-10 as well as Arg-1 (Mantovani et al., 2004), which regulates Th2-type immune responses and promotes tumor growth and metastasis. Additionally, they can induce the expression of vascular endothelial growth factor (VEGF), thus prompting tumor angiogenesis (Mantovani et al., 2017). In response to different stimuli, four subtypes of M2 macrophages are known: M2a, M2b, M2c, and M2d. M2a macrophages are activated by IL-4 and IL-13, while M2b macrophages are activated by Toll-like receptor ligands, and IL-1β. Additionally, M2c macrophages are induced by IL-10, TGF-β and glucocorticoids, while M2d macrophages are induced by IL-6 and adenosine (Rőszer, 2015). While macrophage activation and polarization allow them to adopt specific phenotypes, their inherent plasticity enables these immune cells to shift between different phenotypes (Smith et al., 2017). Metabolic changes in fatty acid and glucose utilization drive macrophage polarization towards the M1 or M2 subtype, thus affecting their plasticity (El Kasmi and Stenmark, 2015). Macrophages are highly adaptable, with polarization occurring along a continuum that encompasses various intermediate phenotypes (Zhang et al., 2022). This plasticity is supported by a responsive epigenome influenced by significant changes in DNA methylation (Li Z. et al., 2024). Such characteristics provide macrophages with heterogeneity and the ability to modify their functional phenotype in response to microenvironmental stimuli (Smith et al., 2017). In diseases like GC, the dysregulation of macrophage phenotypes is believed to contribute to pathogenesis. This functional adaptability holds considerable therapeutic promise, as it can potentially be leveraged to restore equilibrium among different macrophage subsets (Zhang et al., 2022). Consequently, it is essential to investigate the diversity of macrophage phenotypes in the context of GC development and how environmental factors affect the behavior of these immune cells (Zhang et al., 2022). Though M1 and M2 macrophages exhibit distinctive functions in biological activities, they can transform into each other, which presenting a promising therapeutic target for GC.

Recent studies utilizing single-cell RNA sequencing have offered a static transcriptional overview of the diversity of TAMs (Xu et al., 2024). Consequently, new molecular definitions for macrophage states are being applied in immuno-oncology and are being evaluated for their prognostic and predictive significance (Pittet et al., 2022). Based on these findings, the M1-M2 classification of macrophages has faced criticism due to the dynamic nature of macrophages, whose gene expression profiles can shift in response to changes in the microenvironment (Gao et al., 2025). In recent years, TAMs can be categorized into several subtypes, distinguished by their signature genes, signaling pathways, and functional characteristics: interferon-primed TAMs (IFN-TAMs), immune-regulatory TAMs, inflammatory cytokine-enriched TAMs, and proliferative TAMs (Prolif-TAMs) (Ma et al., 2022). Immune regulatory TAMs display characteristics similar to alternatively activated macrophages, which have been identified in a variety of tumors (Wu et al., 2025; Yu et al., 2025). IFN-TAMs are defined by the high expression of interferon-regulated genes, including CXCL10 and ISG15 (Cheng et al., 2021). However, contrary to the common assumption that M1 macrophages possess antitumor properties, IFN-TAMs demonstrate immunosuppressive functions. They have been shown to suppress immune responses through mechanisms such as tryptophan degradation and the recruitment of Tregs (Sadik et al., 2020). Prolif-TAMs exhibit high levels of Ki-67 and cell division cycle 45, which indicates a potential proinflammatory phenotype (Peng C. et al., 2024). However, it remains uncertain whether these cells represent a temporary state that quickly differentiates into other TAM subsets or if they persist as cycling progenitors, which await further investigations.

In the context of GC, the traditional M1-M2 classification has also been challenged. For example, Du et al. revealed the five types of TAM subset marker genes in GC tissues. However, the co-expression of M1 and M2 gene signatures in all five macrophage subtypes indicated that macrophages exhibit a greater complexity, challenging the traditional binary classification of M1 and M2 TAMs (Du et al., 2024). Among them, SPP1 ^+ ^TAMs possessed higher M2 signatures that played an essential immunosuppressive role in the TME of GC (Du et al., 2024). Additionally, the CTSB^+^ macrophages also played a role in sustaining the immunosuppressive environment of GC (Wang Q. et al., 2025). In line with this, Li et al. reported that all TAM clusters did not fit within the M1/M2 dichotomy in GC tissues with peritoneal metastasis. Thus, they divided TAMs into different groups based on diverse expression profiles of CTS and C1Q-associated genes. This suggested that the expression variation of CTS and C1Q genes in ascitic TAMs represented a continuum rather than a binary polarization (Li Y. et al., 2024). Additionally, pathway analyses of single-cell RNA sequencing data have revealed the presence of diverse metabolic processes within TAM clusters (Qian et al., 2024). Furthermore, growing evidence indicates a significant correlation between the phenotypic characteristics and metabolic profiles of TAMs (Qian et al., 2024). These investigations have demonstrated that metabolism can drive the phenotypic and functional changes of TAMs in response to variations in the tumor microenvironment. Moreover, epigenetic changes, such as DNA methylation and histone modification, play a crucial role in defining the characteristics and functions ofTAMs. Investigating this area can aid researchers in understanding how the tumor microenvironment influences the state of TAMs through epigenetic mechanisms, while also exploring potential intervention strategies (Ji et al., 2025). The longitudinal and local heterogeneity of TAM states is now achievable through single-cell and spatial transcriptomics. This comprehensive understanding of macrophage classification is laying the groundwork for a new era of GC treatment, ultimately leading to improved effectiveness of therapeutic strategies.

### 2.2 The crosstalk between TAMs and other immune cells within the TME of GC

TIME is an intricate microenvironment, which possesses various immune cells and non‐malignant mesenchymal cells (Franzese, 2024). In the TIME, TAMs hold a significant position by interacting with other types of immune cells, thus modulating immune response in the context of GC. These communications can influence the predominant type of TAMs, M2 TAMs, through the secretion of various communicative mediators, thereby creating an immunosuppressive microenvironment that is optimal for cancer progression (Li D. et al., 2023; Zhang G. et al., 2023; Su et al., 2022).

#### 2.2.1 TAMs interact with T cells via PD-1 and STING pathway

SIGLEC10 was found to inhibit functional activation and proliferation of tumor-infiltrating CD8^+^ T cells by Akt/P38/Erk cascade in GC. Guo et al. identified the infiltration of SIGLEC10^+^ macrophages in GC tissues via analyzing single-cell RNA sequencing data. Moreover, SIGLEC10^+^ macrophages exhibited M2-like phenotype, which displayed high expression of exhaustion markers such as PD-1, T-cell Immunoglobulin and Mucin-Domain Containing-3 (TIM-3) and CD39, which was also associated with exhaustion of CD8^+^ T cells, proving novel target to improve T cell function for GC therapy (Guo et al., 2023). Moreover, Lin et al. claimed that CSF-2 promoted the M2 macrophage-mediated C-X-C Motif Chemokine Ligand 8 (CXCL8) secretion, leading to upregulated expression of PD-L1 and activation of signal transducer and activator of transcription 3 (STAT3) pathway, which further downregulated tumor-infiltrating CD8^+^ T cells as well as promoted functional exhaustion of T cells, exhibiting unfavorable prognosis in GC patients (Lin et al., 2019). IL-4 mediated M2 macrophage polarization also promoted phosphoinositide 3-kinase (PI3K)/AKT/mechanistic target of rapamycin (mTOR) pathway, resulting in prompt glycolysis and upregulated the expression of Fcγ receptor IIB (FcγRIIB), which ultimately functional dysregulation of CD8^+^ T cells in GC (Zhang J. et al., 2024). Miao et al. reported that the repression of STING pathway and its activation by 2'3'-c-GAMP promoted the polarization of TAMs towards a pro-inflammatory subtype through the IL6R-JAK-IL24 pathway. Additionally, these changes significantly decreased the number of tumor-infiltrating CD4^+^ T cells and increased the CD8^+^/CD4^+^ ratio in GC tumors (Miao et al., 2020).

Existing evidence has demonstrated that human TAMs expressed PD-1. PD-1 expression of TAM was inversely related to the phagocytic ability against tumor cells. Recent studies have revealed that obesity could induce expression of PD-1 in TAMs, which significantly provided negative feedback to TAMs including the suppression of their glycolysis, phagocytosis, and their T cell stimulatory potential (Bader et al., 2024). Moreover, PD-1 deficiency slowed tumor growth, enhanced TAMs glycolysis and antigen-presentation capability, and increased CD8^+^ T cell activity while reducing markers of exhaustion (Bader et al., 2024). Additionally, TAM secreted Itaconate that enhanced the expression of PD-1 by promoting the upregulation of H3K4me3 at the Eomes promoter, thus promoting the tumor evasion by causing CD8^+^ T-cell exhaustion. (Bader et al., 2024). *In vivo* blockade of the PD-1-PD-L1 interaction enhanced macrophage phagocytosis, reduced tumor growth, and prolonged survival in mouse cancer models (Gordon et al., 2017). The prevalence of PD-1^+^ macrophages was notably elevated in GC tissues. In terms of phagocytic activity, PD-1^+^ macrophages exhibited significant impairment compared to their PD-1^−^ counterparts. Furthermore, the frequency of PD-1^+^ macrophages were identified as an independent prognostic factor for patients survival (Kono et al., 2020). Wang et al. identified a specific subset of TAMs that highly expressed PD-1 in GC patients with advanced stages. These PD-1^+^ TAMs displayed M2-like characteristic, could significantly impede CD8^+^ T-cell function by triggering PD-1 signaling pathway. Further mechanistic studies revealed that tumor cell-derived exosomes upregulated the transformation from monocytes into PD-1^+^ TAMs, leading to an immunosuppressive TIME (Wang et al., 2018).

Additionally, TAMs are capable of expressing TIM-3, which could trigger functional inactivation of T cell (Wang et al., 2017). M2 TAMs also promoted the acclamations of lipids in the TIME, thus impairing T cell function (Luo et al., 2020). Furthermore, M1 TAMs enhanced the immune clearance of GC cells by improving the functionality of CD8^+^ T cells through the increased secretion of TNF-α in the TME (Li et al., 2019). M1 TAMs also secreted exosomes contained miR-16-5p that reduced expression of PD-L1 on GC cells, resulting in activated T cells (Li et al., 2020). These findings indicated that targeting these molecules that mediated the functional regulation of CD8^+^ T cells presented a promising immunotherapeutic approach.

#### 2.2.2 TAMs interact with CAFs via targeting functional proteins

CAFs play a pivotal role in shaping the TME of GC, particularly by modulating TAMs polarization. In the context of GC, CAFs were found to highly express insulin-like growth factor binding protein 7 (IGFBP7), which induced the polarization of M2 TAMs via the fibroblast growth factor 2 (FGF2)/FGFR1/PI3K/AKT signaling pathway (Li D. et al., 2023). In line with this, CAFs secreted POSTN to promote M2 TAMs polarization in GC (You et al., 2023). Recently, extracellular vesicles (EVs) secreted by CAFs were found to induce the polarization of M2 macrophages by delivering miR-4253 that directly targeted IL6R (Duan et al., 2024). CAFs also expressed FERMT2 that promoted M2-like TAMs polarization (Yin et al., 2024), suggesting a fibroblast/FERMT2/M2 TAM axis for GC treatment. SPP1^+^ macrophages was a novel type of TAMs that have been identified by single-cell RNA sequencing. Notably, SPP1+ macrophages interacted with FAP-positive CAFs, contributing to the establishment of a hypoxic tumor microenvironment, which is known to promote tumor progression and resistance to therapy (Su et al., 2024).

#### 2.2.3 TAMs interact with MSCs via NF-kB and STAT3 pathway

GC cells secreted exosomes activated mesenchymal stem cells (MSCs) by prompting NF-kB signaling pathway, leading to enhanced TAM functions and supported tumor growth (Shen et al., 2019). Moreover, MSCs derived from GC cells activated the Janus kinase 2 (JAK2)/STAT3 signaling pathway, thereby enhancing the M2 TAMs polarization through increased secretion of IL-6 and IL-8 (Li et al., 2022).

#### 2.2.4 TAMs interact with NK cells and B-lymphocytes via TGF-β pathway

**FIGURE 2:**
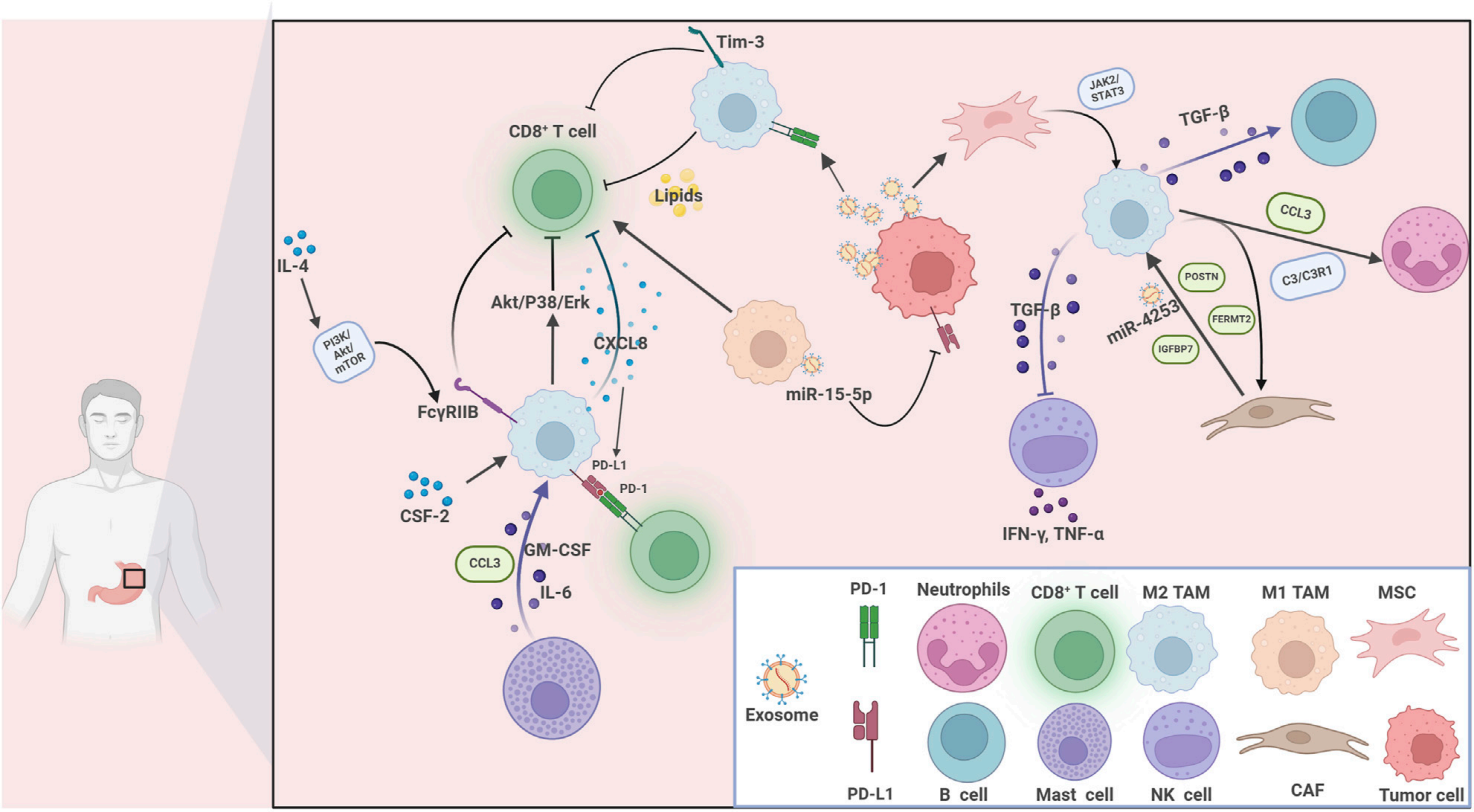
The communications between tumor-associated macrophages and various immune cells within the tumor immune microenvironment in gastric cancer. Immune cells interplay with tumor cells in the gastric cancer TME. Key players include CD8^+^ T cells, M1 and M2 tumor-associated macrophages (TAMs), neutrophils, B cells, mast cells, NK cells, mesenchymal stem cells (MSCs), cancer-associated fibroblasts (CAFs), and tumor cells. Multiple cytokines, chemokines, and signaling pathways mediate these interactions. For example, GM-CSF, IL-6, CXCL8, and CCL3 facilitate cellular communication and recruitment. The PI3K/Akt/mTOR, Akt/P38/Erk, TGF-β/STAT3, and JAK2/STAT3 pathways are involved in immune modulation, cell polarization, and tumor progression. Immune checkpoints (PD-1, PD-L1, Tim-3), exosomes (carrying miR-15-5p, miR-4253), and lipids further contribute to immunosuppression and tumor immune escape. Key molecules such as POSTN, FERMT2, and C3/C3R1 regulate the behavior of stromal and immune cells.

NK cells are a crucial component of the antitumor immune response in GC. Isolated TAMs have the ability to suppress the expression of IFN-γ, TNF-α, and Ki-67 in NK cells. Blocking TGF-β1 alleviated the inhibitory effects of TAMs on the functions of NK cells (Peng et al., 2017). Moreover, Peng et al. depicted dynamics communications within TIME in advanced GC patients that TAMs recruited CXCL2+CAFs and CMTM2+ neutrophils through complement 3/ complement C3a Receptor 1 (C3/ C3AR1) and annexin axis (Peng H. et al., 2024). TAMs also interacted with B-lymphocytes by the TGF-β signaling pathway, while they also secreted CCL3 to enhanced the recruitment of neutrophils (Peng H. et al., 2024). Furthermore, mast cells were activated by IL-11/IL-33 axis, which released macrophage-related factors including GM-CSF, CCL3, and IL-6 that promoted TAMs polarization. These findings indicate that TAMs interact with various cell types within the TIME, collectively influencing the progression of GC (Figure 2). As additional sources of TAMs are identified, their diversity and complexity within the TME will be more extensively elucidated, which may pave the way for GC treatment.

## 3 Functions and mechanisms of TAMs in GC progression

### 3.1 TAMs in tumor proliferation, invasion and metastasis in GC

Emerging evidence has demonstrated that TAMs and cancer cells engage in interactions mediated by various functional proteins and cytokines, which affect cell proliferation, invasion, migration in the setting of GC (Zhang G. et al., 2023; Deng et al., 2024; Piao et al., 2022). Within the hypoxia microenvironment, TAMs were stimulated to secret CXCL8, which significantly prompted cell proliferation and invasion in the cell lines of GC. Further mechanistic investigations have revealed that hypoxia activated C-X-C Motif Chemokine Receptor 1/2 (CXCR1/2), leading to the activation of JAK/STAT1 pathway, which subsequently triggered the secretion of IL-10 to facilitate macrophages polarization towards M2 phenotype, further established a reciprocal loop between TAMs and tumor cells (Piao et al., 2022). Moreover, GC cell-derived exosomes packaged circATP8A1, which sponged miR-1-3p and activated STAT6 cascade, resulting in promoted cell proliferation and metastasis by inducing M2 polarization of TAMs to enhance the secretion of IL-10, TGF-β, and CXCL1 (Deng et al., 2024). Additionally, M2 TAMs secreted EVs packaged apolipoprotein to activate PI3K/Akt signaling pathway, leading to enhanced cell proliferation, invasion and tumor growth in GC (Z et al., 2018). In line with this, TAMs enhanced TNF-α secretion by triggering the TNFR1/ERK/VGLL1 axis, facilitating the cell viability in the setting of GC (Hwang et al., 2022). Moreover, the MIR181A2HG/miR-5680 axis acted as a novel ceRNA pathway promoting versican (VCAN) expression. VCAN, via paracrine signaling, bound CD44 on TAMs to induce M2 polarization and increase VEGF-C secretion, facilitating lymphangiogenesis. Meanwhile, autocrine VCAN binding to CD44 on GC cells activated the Hippo pathway and SP1, further upregulating MIR181A2HG and forming a positive feedback loop that promotes lymphatic metastasis (Zang et al., 2025). Additionally, CEBPB-driven transcription elevated SERPINE1 levels, which, through autocrine signaling, activated PI3K/AKT and EMT pathways to enhance anoikis resistance and metastatic capacity in GC cells. Paracrine SERPINE1 also promoted M2 macrophage polarization via LRP1, leading to reduced CD8^+^ T-cell infiltration and suppressed antitumor immunity. Thus, SERPINE1 is a key mediator of GC progression and immune evasion, representing both a potential therapeutic target (Wang B. et al., 2025). These findings revealed that TAMs play an essential role in cell proliferation and invasion in the context of GC.

**FIGURE 3:**
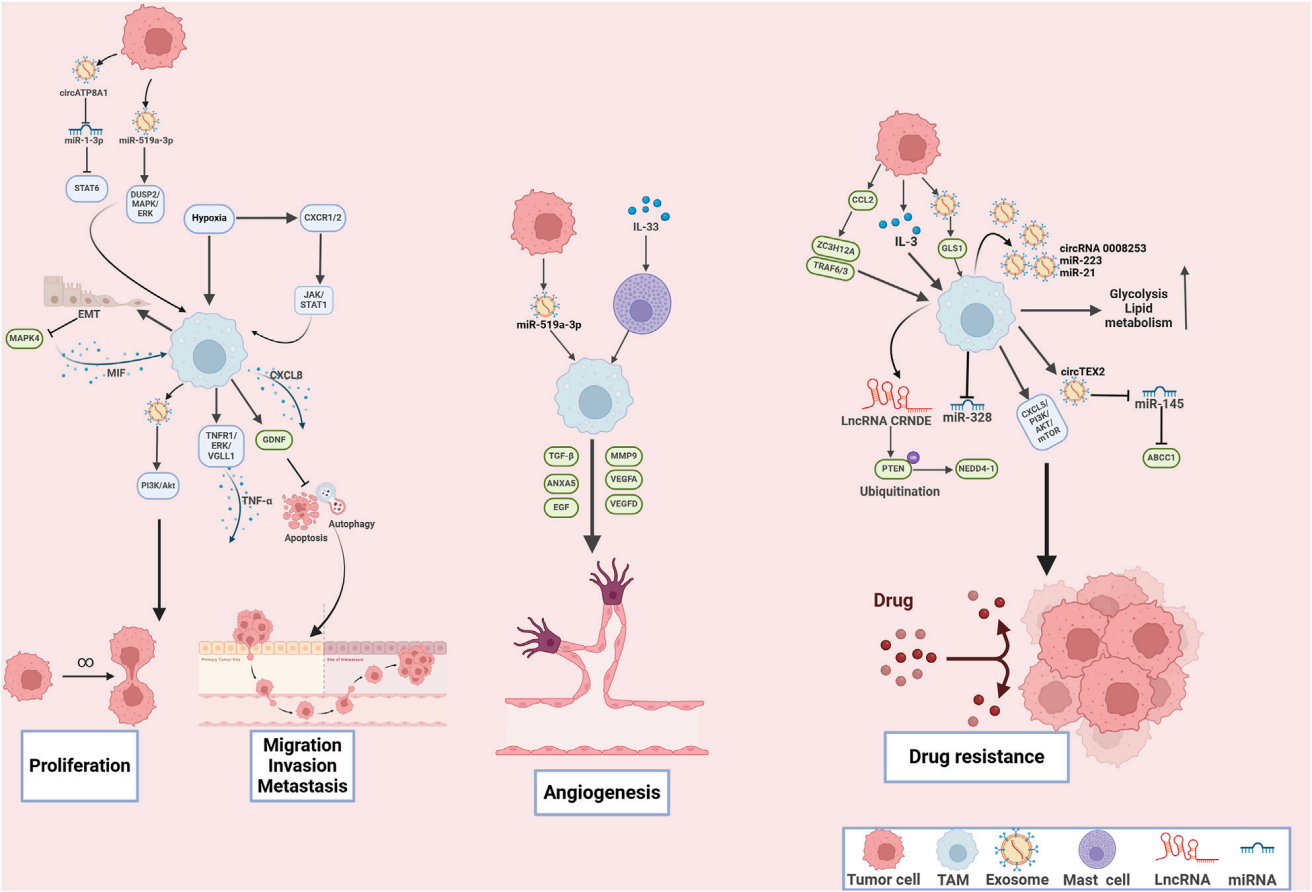
The mechanisms of tumor‐associated macrophages in tumor cell proliferation, metastasis, angiogenesis and drug resistance in gastric cancer. Tumor-associated macrophages play multifaceted roles in driving gastric cancer cell proliferation, metastasis, angiogenesis, and drug resistance through complex molecular interactions and signaling pathways.

Enhanced angiogenesis is the critical process in the formation of the pre-metastatic niche (Liu and Cao, 2016). For example, epiregulin, cyclooxygenase-2, MMP-1, and MMP-2, expressed by cancer cells, collaboratively orchestrate a multifunctional vascular remodeling program that enhanced vascular permeability and drives the progression of lung metastasis (Gupta et al., 2007). Moreover, TAMs are capable of antigen presentation and initiating immune responses, leading to production of various tumor-derived secreted factors (TDSFs), EVs, and other molecular components (Nasrollahzadeh et al., 2020). These substances are released into the bloodstream, and accumulated at the distant site, the microenvironment gradually transforms into a premetastatic niche that is conducive to the colonization and growth of tumor cells (Wang Y. et al., 2024). In GC patients with liver metastasis, serum exosomal miR-519a-3p (exo-miR-519a-3p) was elevated and associated with poor prognosis. Furthermore, exo-miR-519a-3p activated the mitogen-activated protein kinase (MAPK)/ERK signaling pathway via dual specific phosphatase 2 (DUSP2), leading to M2-type polarization of TAMs. These changes promoted angiogenesis, leading to formation of intrahepatic premetastatic niches and further facilitated the liver metastasis (Qiu et al., 2022). Li and colleagues further expanded the molecular mechanisms on liver metastasis of GC. Their study indicated that TAMs activated epithelial-mesenchymal transition (EMT) to suppress MAPK4 expression, resulting in enhanced secretion of macrophage migration inhibitory factor (MIF), which further polarized TAMs towards M2 phenotype. The M2 TAMs-mediated MAPK4 downregulation facilitated liver metastasis, which might provide novel strategy for GC treatment (Li S. et al., 2023). TAMs transcriptionally increased the expression of glial cell-derived neurotrophic factor (GDNF) in GC, which formed GDNF/ GDNF family receptor alpha 1 (GFRA1) axis to modulate lysosomal functions, leading to inhibited cell apoptosis and autophagy flux, leading to enhanced liver metastasis under the metabolic stress (Ni et al., 2023). Additionally, emerging evidence has further exploited molecular mechanism underlying the tumor-promoting role of TAMs in GC cell proliferation, growth, invasion and metastasis (Eum et al., 2020; Gao et al., 2024; Ito et al., 2021; Shen et al., 2013; Tang et al., 2021; Wang et al., 2020; Xiao et al., 2024; Yang et al., 2021; Yao et al., 2023; Zhang S. et al., 2023), which highlight that targeting TAMs may be promising strategy for GC treatment (Figure 3). In conclusion, TAMs play an essential role in GC cell proliferation and metastasis, which may provide potential therapeutic targets.

### 3.2 TAMs in the angiogenesis in GC

Angiogenesis is a multifaceted process that is crucial for the growth and development of organs, the regeneration of tissues, and the progression of various pathological conditions including cancer (Giubelan et al., 2023). Research has demonstrated that TAMs contribute to tumor angiogenesis by secreting various pro-angiogenic factors, including VEGF-A, epidermal growth factor (EGF), TNF-α, and CXCL12 (Martin and Gurevich, 2021). For example, exosomal miR-519a-3p-devried from GC cells that promoted M2-like macrophage polarization, leading to upregulated levels of VEGFA, VEGFD, TGF-β,thus promoting angiogenesis (Qiu et al., 2022). ANXA5 was identified as the TAM-related gene that promoted angiogenesis in GC (Hong et al., 2024). IL-33/mast cells mediated TAM polarization significantly promoted tumor angiogenesis in GC (Eissmann et al., 2019). Moreover, the expression of cyclooxygenase-2 (COX-2) in TAMs played a crucial role in regulating angiogenesis. Recent studies indicated that when co-cultured with GC cells, M2-polarized TAMs secreted matrix metalloproteinase 9 (MMP9) as a result of COX-2 upregulation, thereby facilitating tumor angiogenesis (Oshima et al., 2011). Several studies also demonstrated the stimulatory effects of M2 TAMs on angiogenesis in the setting of GC (Tang et al., 2021; Guo et al., 2022; Mu et al., 2021), suggesting the essential role of TAMs in angiogenesis. Moreover, SPP1+ macrophages interacted with FAP+ CAFs, and endothelial cells through regulating VEGF pathway, ECM remodeling, and hypoxia pathway, collectively facilitating angiogenesis in GC (Su et al., 2024). The aforementioned research demonstrated that TAMs play an essential role in tumor angiogenesis, collectively influencing the progression of GC and severing as promising anti-angiogenesis targets.

### 3.3 TAMs in drug resistance in GC

During the treatment with chemotherapeutic drugs, TAMs were recruited and polarized to the M2-like phenotype (Ishimoto et al., 2014). M2 TAMs increased CD44 expression by suppressing miR-328, thereby promoting cancer progression through enhanced ROS defense and resistance to chenotherapy (Ishimoto et al., 2014). Oxaliplatin, the third-generation platinum-based chemotherapeutic agent, sever as first-line anti-cancer drug GC (Lordick et al., 2014). However, resistance to oxaliplatin significantly worsened clinical prognosis (Lordick et al., 2014). M2-polarized TAMs secreted exosomes (M2-Exos), which could transfer circRNA 0008253, miR‐223 and miR-21 from TAMs to tumor cells, resulting in reduced cell apoptosis and enhanced resistance to oxaliplatin, doxorubicin, and cisplatin (Yu D. et al., 2023; Gao et al., 2020; Zheng et al., 2017). The transfer of exosomal circTEX2 from M2 TAMs to tumor cells significantly promoted cisplatin resistance via sponging miR-145 and indirectly targeting ATP Binding Cassette Subfamily C Member 1 (ABCC1) (Qu et al., 2024). TAMs also secreted EVs packaged long noncoding RNA (LncRNA) CRNDE, which facilitated the ubiquitination-mediated degradation of phosphatase and tensin homolog (PTEN) and activating the neural precursor cell expressed developmentally downregulated protein 4-1 (NEDD4-1) axis, thus increased resistance to cisplatin in the cell lines of GC (Xin et al., 2021). Moreover, 5-fluorouracil (5-FU) continues to be the first-line chemotherapeutic agent for gastric cancer; however, the development of chemoresistance often leads to unsatisfactory clinical outcomes (Liu J. et al., 2024). During the treatment with chemotherapeutic drugs, TAMs were recruited within the TME. TAMs conferred 5-FU chemoresistance to GC cells by activating CXCL5/PI3K/AKT/mTOR signaling pathway (Su et al., 2022). Furthermore, GC cells secreted IL-3 to induce a shift in macrophage polarization towards a M2-like phenotype, which further promoted GLUT3-mediated glycolysis reprogramming to secret CCL8 for activating JAK/STAT pathway that promoted 5-FU resistance (He et al., 2021). Moreover, GC patients with peritoneal metastasis (GCPM) frequently exhibit a rapidly declining clinical course marked by chemotherapeutic resistance and dismal prognosis (Li Z. et al., 2023). Single-cell RNA sequencing analysis revealed that C1Q^+^ macrophages infiltrated in the TME of GCPM therapeutic failure cases, which exhibited high M2_scores. C1Q^+^ macrophages might contribute chemotherapeutic resistance through enhancing lipid metabolism activity, glycolytic activity and activating the complement and annexin pathways to interplay with CAFs and neutrophils within the TME, providing an intracrine molecular network that could be targeted for GCPM treatment (Peng H. et al., 2024).

Trastuzumab serves as a primary targeted treatment for GC patients with positive human epidermal growth factor receptor-2 (HER2) expression, which has significantly impeded clinical efficacy due to drug resistance (Malla et al., 2024). Tumor cells produced microvesicles packaged glutaminase 1 (GLS1) via cell division cycle 42 (CDC42) that promoted M2 TAMs polarization and contributed to trastuzumab resistance (Hu et al., 2023). Moreover, tumor cells enhanced the expression of CCL2 that impaired M1 macrophages polarization via activating the transcription factor ZC3H12A and regulating TRAF6/3, thus promoting trastuzumab resistance (Sun et al., 2022). These findings revealed that targeting TAMs and disrupting the interactions between TAMs and tumor cells could be promising therapeutic approaches for overcoming drug resistance in GC patients.

### 3.4 TAMs in the immunotherapy resistance of GC

Recent advancements in immune checkpoint inhibitors (ICIs) have shown significant potential in the treatment of GC. Nevertheless, only a small proportion of GC patients exhibit durable responses to treatment with PD-1 or PD-L1 inhibitors. This limited efficacy may be attributed to the heterogeneity present within the TIME (Yasuda and Wang, 2024), as well as resistance to immunotherapy (Liu et al., 2023). In the TME, high levels of macrophage infiltration are often strongly associated with resistance to PD-1/PD-L1 immune checkpoint inhibitors (Kim et al., 2020). TAMs could secrete TGF-β to alter PD-L1 expression. For instance, TGF-β induced PD-L1 expression in tumor cells and enhanced angiogenesis, potentially through succinate accumulation in tumor cells (Gómez et al., 2020). Additionally, TAMs increased PD-L1 expression in tumor-infiltrating myeloid cells via the COX-2/mPGES1/PGE2 pathway, leading to the exclusion of CD8^+^ T cells (Prima et al., 2017). Additionally, researchers have observed IL-4 induced glycolysis in TAMs by activating the PI3K/AKT/mTOR signaling pathway. Ultimately, these changes led to functional impairment of CD8^+^ T cells in GC and contribute to resistance to PD-1 antibody therapy (Zhang J. et al., 2024).

#### 3.4.1 TAM-mediated immunotherapy resistance via PD-L1/CXCL8

Additionally, emerging evidence has revealed that TAMs play an essential role in immunotherapy resistance (Shi B. et al., 2024). Comprehensive biomarker profiling based on REGOMUNE phase II study has demonstrated that M2-like macrophages were significant abundance in the TIME of GC (Cousin et al., 2024). Further mechanistic studies have revealed that M2 macrophage-derived exosomes (M2-Exos) upregulated PD-L1, leading to inhibited T cell activation and proliferation, thus contributing to resistance to ICIs (Wang et al., 2021). TAMs secreted CXCL8 to upregulate PD-L1 on TAMs, thus determining immune evasion and resistance to ICIs (Lin et al., 2019). Moreover, TAMs also promoted ICI resistance via CXCL16-CXCR6. For example, TAMs also recruited CD8^+^ T cells that solely expressed NKG2A via the CXCL16-CXCR6 pathway, leading to anti-PD-1 resistance in GC patients (Li G. et al., 2024), supporting the potential of NKG2A as a novel immunotherapeutic target and present fresh perspectives on combination strategies involving ICIs in GC (Li G. et al., 2024). These evidence has revealed that TAMs could promoted immunotherapy resistance by regulating PD-L1/ CXCL8 and CXCL16-CXCR6 pathway.

In GC, Quiescent cancer cells (QCCs) were found to promote the glycolytic reprogramming of M2 macrophages, which promoted hypoxia inducible factor 1 alpha (HIF1A)-mediated T-cell exhaustion and conferred cell resistance to ICIs (Qi et al., 2024). Moreover, melatonin significantly enhanced the production of cancer-derived exosomes and releasing exosomal miRNAs, which significantly enhanced anti-tumor immunity. Mechanistically, exosomal miR-20b-5p, miR-17-5p, and miR-93-5p transcriptionally and translationally reduced PD-L1 levels in TAMs, which facilitated the infiltration of CD8^+^ T cells into the tumor site and boosted anti-tumor immune responses (Wang et al., 2023). Furthermore, GC cells secreted legumain upon binding to the integrin αvβ3 on the cell surface, subsequently activating the αvβ3/PI3K/AKT/mTORC2 signaling pathway. This activation promoted metabolic reprogramming and facilitated the polarization of macrophages from the M1 phenotype to the M2 phenotype, leading to anti-PD-1 immunotherapy resistance (Pei et al., 2024). MUC1^+^ GC cells secreted growth differentiation factor 15 (GDF15) to facilitate the immunosuppressive functions of M2 TAMs, which in turn influenced the functions of T lymphocytes via the SPP1-CD44 interaction, thus promoting immunotherapy resistance (Peng H. et al., 2024). IL-4 skew TAM polarization to M2-like phenotype, which further enhanced FcγRIIB expression and promoted PI3K/AKT/mTOR axis, resulting in immune resistance to anti-PD-1 treatment (Zhang J. et al., 2024). Dickkopf-1 (DKK1) mediated the induction of M2 TAMs impaired the clinical effacicy of PD-1 antibody treatment by interplaying with cytoskeleton-associated protein 4 (CKAP4) and facilitating PI3K-AKT pathway (Shi et al., 2022). Through the exploration of these complex interactions, these findings deliver important insights into precision medicine and aims to improve therapeutic outcomes in the treatment of GC.

## 4 Therapeutic interventions of TAMs in GC

An increasing body of evidence is enhancing our perspectives on TAMs in the tumorigenesis of GC. TAMs play an essential role in cell proliferation, invasion, migration and drug resistance of GC, which hold immense potential in cancer therapy. There are various strategies specifically designed to target macrophages in the preclinical and clinical settings, which including targeting TAM infiltering and macrophage polarization. Additionally, engineered macrophages such as chimeric antigen receptor macrophages (CAR-M) have progressed into clinical evaluation, which demonstrated promising results in GC treatment (Table 1).

**TABLE 1:** Therapeutic interventions of tumor‐associated macrophages in gastric cancer.

Therapeutic strategy	Inhibitor	Mechanisms	Outcome	Clinical relevance	References
Inhibition of M2 TAM infiltration	PAS monotherapy or its combination with PD-1 antibody	Decreased the number of M2-type TAMs	Inhibited tumor growth	Preclinical	Smith et al. (2021)
Inhibition of M2 TAM infiltration	CD40×HER2 bispecific antibody	Restored the TRAF6/3 ubiquitination and activated NF-κB signaling pathway	Decreased infiltration of M2 TAMs. Improved anti-tumor effects and reversed trastuzumab resistance in HER2-positive GC.	Preclinical	Sun et al. (2022)
Inhibition of M2 TAM infiltration	Photodynamic therapy with M-chlorin	Induced apoptosis of TAMs	Inhibited tumor growth by promoted TAMs and tumor cell apoptosis	Preclinical	Hayas et al. (2015)
Inhibition of M2 TAM infiltration	Modified Jianpi Yangzheng	Reduced the transfer of exosomal PKM2 from tumor cells to TAMs, mitigating the infiltration of M2 TAMs	Inhibited tumor growth	Preclinical	Wu et al. (2022)
Repolarization of TAMs	IU1(USP14 inhibitor)	Blocked USP14- SIRT1 axis	Induced the repolarization of TAMs from the M2 phenotype to the M1 phenotype, thereby enhancing anti-tumor activity	Preclinical	He et al. (2023)
Repolarization of TAMs	PI3K-γ inhibitor (IPI-549)	Restored phagocytosis and the polarization of M2-type TAMs	Inhibited polarization of M2 TAMs and enhanced anti-tumor activity	Preclinical	Luo et al. (2020)
Repolarization of TAMs	DNase-1(neutrophil extracellular trap degrader)	Reduced the effects of TREM1-induced polarization of M2 macrophages	Inhibited polarization of M2 TAMs and suppressed tumor growth	Preclinical	Yu et al. (2024)
Repolarization of TAMs	DKN-01(DKK1 inhibitor)	Promoted cGAS/STING pathway by specifically targeting DKK1	Inhibited polarization of M2 TAMs and suppressed tumor growth	Preclinical	Yang X. et al. (2024)
Repolarization of TAMs	Methionine restriction	Suppressed MIF derived from tumor cells	Enhanced the M1-type polarization and suppressed tumor growth	Preclinical	Xin et al. (2025)
Repolarization of TAMs	Opioid-free anesthesia	Promoted the convention of M2 TAMs into M1 TAMs	Reduced immunosuppression in the treatment of immunotherapy	Preclinical	Liu W. et al. (2024)
Repolarization of TAMs	Barasertib-HQPA(Aurora kinase inhibitors)	Promoted cellular senescence, thus upregulating MCP-1/CCL2	Induced macrophage to M2-type polarization	Preclinical	Yang R. et al. (2024)
Repolarization of TAMs	Epigallocatechin gallate extracted from Hedyotis diffusa Willd injection	Interacted with STAT3, leading to its reduced nuclear localization, which promoted transcriptional suppression of PLXNC1	Promoted repolarization of M2 TAMs induced by exosomal miR-92b-5p, leading to diminished tumor progression	Preclinical	Yi et al. (2024)
Repolarization of TAMs	Qingrexiaoji Recipe	Converting the M2-type TAMs into M1-type TAMs via modulating miR-29a-3p/HDAC4 axis	Exhibited tumor-suppressive functions	Preclinical	Zhang Y. et al. (2024)
Repolarization of TAMs	Phosphorylated Rehmannia polysaccharide extracted from Rehmannia	Suppressed the LGR6 mediated activation of Wnt/β-catenin pathway	Induced macrophage polarization towards M1 phenotype and inhibited tumor progression	Preclinical	Liu X. et al. (2024)
Repolarization of TAMs	Dendrobium officinale polysaccharides	Modulated JAGGED1/NOTCH1 and STAT6/PPAR-r cascade	Promoted the repolarization of M2 macrophages into M1 macrophages	Preclinical	Zhao et al. (2023)
Repolarization of TAMs	Diosmetin	Interfered with TRAF2/NF-κB pathway	Diminished M2-type TAM polarization	Preclinical	Zhang and Luo (2023)
Repolarization of TAMs	Sophoridine	Regulated TLR4–IRF3 axis	Reduce CD8^+^ T cell exhaustion as well as converting M2-type into M1-type TAMs through	Preclinical	Zhuang et al. (2020)
Repolarization of TAMs	Palmitic acid and γ-IFN	Regulated TLR4 pathway	Increased polarization of M1-type macrophages and impeded M2-type macrophages polarization, thus suppressing tumor progression	Preclinical	Zhang YY. et al. (2023)
Repolarization of TAMs	Calcitriol	Inhibited glycolysis by regulating mTOR pathway	Impeded M2 macrophage polarization and suppressed tumor growth	Preclinical	Jie et al. (2024)
Repolarization of TAMs	Betulinic Acid	Regulated the GRP78-TGF-β1 signaling Pathway ization	Inhibited stemness of cancer cells and inhibited M2 TAM polarization	Preclinical	Chen et al. (2023)
Repolarization of TAMs	Flavokawain B	RegulatedTGF- *β* 1/SMAD4 Pathway	Attenuates M2 TAM Polarization	Preclinical	Zhu et al. (2022)
Repolarization of TAMs	Dextran Sulfate	Repressing the IL-6/STAT3 signaling pathway	Inhibits Angiogenesis and Invasion and impaired polarization of M2 TAMs	Preclinical	Guo et al. (2022)
Combination therapeutic strategies	mAb04-MICA(αVEGFR2–MICA fusion antibodies) plus anti-PD-1 antibody	Exhibited great synergistic effects with anti-PD-1 therapy by repolarization of TAMs	Impeded M2 macrophage polarization and suppressed tumor growth	Preclinical	Pan et al. (2023)
Combination therapeutic strategies	OBP-702(p53-expressing oncolytic adenovirus) plus anti-PD-1 antibody	Exhibited great synergistic effects with anti-PD-1 therapy by repolarization of TAMs	Suppressed peritoneal metastasis	Preclinical	Tabuchi et al. (2024)
CAR-M	HF-CAR-M	Targeting HER2 pathway	Suppressed tumor growth	Preclinical	Dong et al. (2023)

### 4.1 Inhibiting the infiltration of M2 TAMs in TME

Given the essential role of TAMs in the progression of GC, eliminating TAMs within the TIME could benefit GC patients. Gastrointestinal peptide gastrin has been reported to activate the function of TAMs by increasing the expression of IL-1β, IL-3Rβ, and SDF-1α, which fostered the formation of TME (Han et al., 2013). Moreover, gastrin has been demonstrated to facilitate tumor growth via paracrine and autocrine mechanisms mediated by the cholecystokinin-B receptor (CCK-BR), which has been found to be expressed in approximately 50% of GC patients (Duan et al., 2022). Polyclonal antibody stimulator (PAS), a specific immunogen vaccine that could stimulate antibodies targeting gastrin peptide, thus impeding GC progression. Research performed by Simth et al. reported that either PAS monotherapy or its combination with PD-1 antibody led to a decrease in the number of M2-type TAMs (Smith et al., 2021). High expression of CCL2 was found to be associated with increased the proportion of M2-like phenotype macrophages, which could be attributed to activated ZC3H12A-TRAF6/3 axis, which could be reversed by CD40-HER2 bispecific antibody that recovered the ubiquitination process of TRAF6/3, leading to decreased M2-like TAMs in the TIME of HER2-positive GC (Sun et al., 2022). Mannose-conjugated chlorin (M-chlorin), a specific type of photosensitizer that could effectively bind to mannose receptors, which are prominently expressed on TAMs. Photodynamic therapy with M-chlorin were demonstrated to induce effectively cell apoptosis of TAMs and tumor cells within the TME, thus blocking GC progression (Hayas et al., 2015). Modified Jianpi Yangzheng (mJPYZ), an empirical decoction derived from Traditional Chinese medicine has been found to significantly increase the survival duration of patients suffering from advanced-stage GC. Mechanistically, mJPYZ reduced the transfer of exosomal pyruvate kinase M2 (PKM2) from GC cells to macrophages, mitigating the infiltering of M2-type TAMs within the TIME, thereby ultimately impairing tumorigenesis in GC experimental models (Wu et al., 2022). These findings reveal that mJPYZ, PAS and M-chlorin function as essential therapeutic agents for GC treatment by inhibiting the infiltration of M2-like TAMs. Although significant progress has been made in preventing tumor progression by inhibiting the recruitment and activity of TAMs. However, it is crucial to accurately assess both the extent and duration of TAM depletion. An excessive reduction in macrophage levels may inadvertently promote tumor progression (Bercovici et al., 2019). Increasing the drug dosage to deplete TAMs can also lead to a higher incidence of adverse events. Consequently, this technique requires further clinical exploration to mature and establish its safety and efficacy. Future research should focus on addressing these issues, thus paving the way for GC treatment.

### 4.2 Repolarization of macrophages

Mounting experimental data indicate that targeting the polarization of TAMs may be advantageous in cancer immunotherapy (Anderson et al., 2021). Researchers are increasingly utilizing the plasticity of macrophages to enhance polarization towards M1 phenotype or inhibit polarization towards M2 phenotype (Zhang and Sioud, 2023). Ubiquitin-specific protease 14 (USP14) served as an immunosuppressive factor in GC and associated with the polarization of M2 TAMs. The activation of USP14 stabilized SIRT1, which played an essential role in the fatty acid oxidation of TAMs. Blocking USP14- SIRT1 axis by IU1, the USP14 inhibitor, significantly impeded macrophage polarization towards M2 phenotype, thus inhibited tumor metastasis (He et al., 2023). The PI3K-γ inhibitor (IPI-549) was found to restore phagocytosis and polarization of M2 TAMs that were suppressed by lipid accumulation, thereby enhancing anti-tumor responses (Luo et al., 2020). In line with this, neutrophil extracellular trap (NET) degrader DNase-1 mitigated the effects of triggering receptor expressed on myeloid cell 1 (TREM1)-induced M2 macrophage polarization (Yu et al., 2024). Dickkopf-1 (Dkk1) functions as an inhibitor of Wnt signaling pathway through a negative regulatory mechanism, which is associated with poor prognosis in patients with various types of cancers (Wall et al., 2020). DKN-01, the specific antibody that targets DKK1, has been reported to inhibit GC progression by promoting M1 polarization and suppressing M2 polarization of TAMs via promoting the activation of cGAS/STING signaling pathway (Yang X. et al., 2024). Methionine restriction enhanced the M1-type polarization via suppressing MIF derived from tumor cells, highlighting a novel avenue for developing therapeutic drugs for GC (Xin et al., 2025). Additionally, Opioid-free anesthesia promoted the convention of M2 TAMs into M1 TAMs, thus reducing perioperative immunosuppression, which provides immense potential for GC treatment (Liu W. et al., 2024). Aurora kinase inhibitors Barasertib-HQPA and Danusertib could promoted cellular senescence, thus upregulating MCP-1/CCL2, ultimately inducing macrophage to M2-type polarization (Yang R. et al., 2024), indicating that clinicians should consider a sequential therapy involving senescent cell clearance following aurora kinase inhibitors treatment. Currently, the CD47-SIRPα axis is being extensively studied within the “do not eat me” signaling pathways (Jia et al., 2021). CD47 levels are consistently elevated across various tumor types and play a crucial role in inhibiting cellular function by binding to the transmembrane protein SIRPα on phagocytic cells (Jia et al., 2021). Furthermore, IHC analysis of GC samples demonstrated a positive correlation between CD47 expression and the infiltration of TAMs. The CD47 antibody increased the phagocytic activity of TAMs and enhanced secretion of IFN-β in Epstein–Barr virus-associated GC (Duan et al., 2023). These findings are paving the way for the clinical translation of TAMs as therapeutic targets.

Natural products and their extracts have a rich history of extensive use in the treatment of cancers (Wang Z. et al., 2024). Yi et al. found that epigallocatechin gallate (EGCG) extracted from Hedyotis diffusa Willd injection (HDI), significantly interacted with STAT3, leading to its reduced nuclear localization. These changes promoted the transcriptional suppression of PLXNC1, resulting in repolarization of M2 TAMs induced by exosomal miR-92b-5p derived from tumor cells, ultimately leading to diminished tumor progression in GC (Yi et al., 2024). In addition, Zhang and colleagues revealed that Qingrexiaoji Recipe, the traditional Chinese medicine, exhibited tumor-suppressive functions on GC cells by converting the M2-type TAMs into M1-type TAMs via modulating miR-29a-3p/HDAC4 axis (Zhang Y. et al., 2024). Additionally, rehmannia polysaccharide, dendrobium officinale polysaccharides, and diosmetin have been demonstrated to regulate M2-type TAM polarization (Liu X. et al., 2024; Zhao et al., 2023; Zhang and Luo, 2023).

Toll-like receptors (TLRs) are crucial for the activation of macrophages and the regulation of immune responses (Gallego et al., 2011). TLR4, the most prominent pattern recognition receptors involved in innate immunity, affecting malignant progression of human tumors (Shetab Boushehri and Lamprecht, 2018). Sophoridine has been shown to reduce CD8^+^ T cell exhaustion as well as converting M2-type into M1-type TAMs through toll-like receptor 4 (TLR4)–interferon regulatory factor 3 (IRF3) axis (Zhuang et al., 2020). The dual combination application of Palmitic acid and γ-interferon(γ-IFN), the TLR4 agonists, significantly increased M1-type macrophages and impeded M2-type macrophages polarization, thus suppressing GC progression (Zhang YY. et al., 2023). Furthermore, emerging studies have revealed that Calcitriol (Jie et al., 2024), Betulinic Acid (Chen et al., 2023), Flavokawain B (Zhu et al., 2022), Dextran Sulfate (Guo et al., 2022) have been involved in the regulation of M1/M2 macrophages polarization, thus providing novel therapeutic strategies for GC treatment. However, this therapeutic strategy encounters several challenges. Strategies aimed at inhibiting M2 TAMs may also affect other immune cell types, leading to alterations in the overall immune response. This could weaken the body’s ability to combat infections and other diseases. Even if M2 TAMs are successfully inhibited, tumor cells may develop resistance through alternative mechanisms, such as acquiring a more aggressive phenotype or modifying their microenvironment, allowing them to continue growing and metastasizing (Tan et al., 2025). Based on these preclinical evidence, more clinical trials are needed to pave the way for the development of TAMs as therapeutic targets in clinical translation.

### 4.3 Combination therapeutic strategies involved TAMs for GC

Significant clinical benefits have been observed with antiangiogenic therapy in multiple types of human cancers. However, the effectiveness of these therapies is hindered by the immunosuppressive tumor microenvironment. Pan et al. developed mAb04-MICA, a fusion antibody that targeting VEGFR2 that is linked to the α1-α2 ectodomain of MHC class I chain-related molecules A (MICA), which exhibited great synergistic effects with anti-PD-1 therapy in GC by repolarization of TAMs (Pan et al., 2023). Furthermore, OBP-702, the p53-expressing oncolytic adenovirus has exhibited significant impacts on the repolarization of TAMs by the combination of anti-PD-1 antibody, leading to suppressed peritoneal metastasis of GC (Tabuchi et al., 2024). Additionally, gastrin vaccine, both as a standalone treatment and in combination with the PD-1 antibody, inhibited GC progression through the reduction of M2 TAMs within the TIME (Smith et al., 2021). The aforementioned facts suggest that targeting TAMs, either alone or in conjunction with ICIs, could represent a promising therapeutic strategy for GC, which await further investigations.

## 5 CAR-M in GC therapy: advancements and challenges

The application of CARs to macrophages represents a promising strategy for enhancing cellular immunotherapy against solid tumors (Liu Y. et al., 2024). CAR-M represents an innovative form of immunotherapy that involves genetically modifying macrophages to express a specific CAR, which enhances the phagocytic activity and antigen presentation of macrophages, leading to greater specificity in targeting tumors and increased anti-tumor effectiveness (Liu Y. et al., 2024; Shi Y. et al., 2024). CAR-M demonstrates enhanced migratory and infiltrative capabilities by producing matrix metalloproteinases, which can degrade the ECM, allowing it to overcome the challenges posed by dense extracellular matrices (Zhang et al., 2019; Liu et al., 2022). By modulating the composition of the extracellular matrix and reducing immunosuppressive factors, CAR-M can enhance the activity and efficacy of other immune cells, such as T cells and NK cells (Maalej et al., 2023). Additionally, it possesses the ability to modulate the phenotype of TAMs (Noy et al., 2014; Lee et al., 2013). Small molecule inhibitors typically target specific signaling pathways to suppress tumor cells; however, they often lack selectivity, potentially affecting healthy cells, and require continuous administration to maintain efficacy, leading to drug resistance (Dong et al., 2021). Additionally, these inhibitors typically do not adapt to changes in the tumor microenvironment, resulting in diminished effectiveness in certain scenarios (Dong et al., 2021). In contrast, CAR-M demonstrates greater adaptability by responding to the biological characteristics and microenvironment of tumors, offering enhanced flexibility. Antibody-based therapies, such as monoclonal antibodies, possess high specificity and can directly target tumor cells or influence signaling pathways (Zinn et al., 2023). Nevertheless, their anti-tumor effects are frequently compromised by immunosuppression within the tumor microenvironment, limiting their efficacy (Zinn et al., 2023). Moreover, unlike CAR-M, which can modulate the local immune environment and alter the tumor microenvironment, antibody therapies generally focus on single antigens, resulting in lower adaptability to various tumor types.

Macrophages can be precisely modified through modern techniques. Zheng et al. performed bioinformatics analyses, which revealed that c-MET was elevated in pancreatic cancer tissues. Furthermore, CAR-M-c-MET cells were developed and exhibited specific binding to cancer cells, which significantly enhanced the phagocytic and cytotoxic capabilities of macrophages and resulted in remarkable tumor suppression in the murine models (Zheng et al., 2024). Although great advancements of CAR-M have been made in solid tumors, there are few studies that have investigated CAR-M based treatment in the setting of GC. Dong and colleagues created a novel CAR-M by genetically modifying macrophages to express a HER2-FcεR1γ-CAR (HF-CAR), which specifically targeted HER2-positive GC and exhibited great synergistic effects in combination with oxaliplatin, leading to significant anti-tumor functions in mouse models (Dong et al., 2023).

Although CAR-M represents a promising approach in cancer immunotherapy, it encounters several challenges, particularly regarding the limited availability of cells and their low proliferation capacity *in vivo*, which can adversely affect treatment efficacy (Li J. et al., 2024). Additionally, macrophages exhibit a lower propensity to circulate in the bloodstream, making the collection and development of these cells a significant challenge in CAR-M production (Shi Y. et al., 2024). Additionally, the heterogeneity of human macrophages in contrast to mouse macrophages, along with the limited understanding of human macrophage biology, presents further difficulties in the production of CAR-M (Wang et al., 2022). On the other hand, due to the multifunctional role of macrophages in immune regulation, patient safety and treatment outcomes may have different implications (Locati et al., 2020; Klichinsky et al., 2020). Research has shown that macrophages play a crucial role in the onset of cytokine release syndrome (CRS) associated with CAR-T therapy, primarily through the production of IL-6, IL-1, and NO (Giavridis et al., 2018; Huang et al., 2024b). Given that CAR-M has the potential to profoundly alter immune system dynamics, the long-term effects of CAR-M are particularly contentious. With advancements in research and the implementation of various innovative solutions, these concerns can be alleviated and CAR-M would be regarded as a promising therapeutic approach in GC.

## 6 Conclusions and perspectives

TAMs function as the essential immunomodulators within the TME of GC. TAMs establish a complex communication network with other types of immune cells or stromal cells, thus contributing to the immunosuppressive TME in GC. Moreover, the crosstalk between TAMs and tumor cells significantly impacts GC progression including tumor growth, metastasis, angiogenesis and drug resistance. As one of the most abundant cell types in solid tumors, TAMs contribute to increased PD-L1 expression in tumor cells and other immunosuppressive cells through the secretion of cytokines. Furthermore, the regulation of the epigenetic state of TAMs, influencing gene expression and thereby enhancing their immunosuppressive functions, which is crucial for the ICI resistance (Zhang and Jagannath, 2025). This understanding not only sheds light on the biological functions of TAMs but also presents potential targets and strategies for future therapeutic interventions.

Besides, the intricate immunomodulatory axis remarkably promotes immunotherapy resistance, thus indicating that targeting TAMs hold immense therapeutic potential for GC patients. Strategies such as eliminating TAMs, repolarization of TAMs, and CAR-M has been verified in multiple studies. Nevertheless, introduction of suitable therapeutic strategies targeting TAMs presents several challenges due to the heterogeneity and complexity of macrophage. Firstly, current studies on TAMs are conducted in the experimental mouse models, which typically exhibit notable differences from the pathogenesis and therapeutic responses observed in humans (Sica and Mantovani, 2012). Studies focusing on the development of feasible inhibitors targeting human TAMs within the TIME of GC are critical for TAM-based treatment. Furthermore, the diversity and plasticity of TAMs in human tumors are significantly evident by the distinct characteristics of TAMs at different stages of carcinogenesis, suggesting that relying on a single specific blocking antibody might not effectively eliminate tumors (Nasir et al., 2023). Therefore, investigating the diversity of TAMs and their developmental relationships with tumor progression at the single-cell level could yield valuable insights into approaches for selectively targeting specific subpopulations, which could be applicable in the clinical settings for GC treatment (Ma et al., 2022). In addition, emerging strategies targeting TAMs such as CAR-M and developing specific inhibitors could remove TAMs within TME, which might obstruct the intrinsic communication among TAMs and other types of cells, leading to dysregulated immune system dynamics. These changes might result in unforeseen toxicities, which await further investigation in the clinical settings. And the elimination of TAMs could promote the compensatory emergence of other types of immunosuppressive cells. More basic studies should in-depth explore the molecular mechanism underlying the role of TAMs in GC, which might pave the way for developing feasible strategies targeting TAMs, ultimately improving the prognosis of GC patients.
